# Adolescent administration of Δ^9^-THC decreases the expression and function of muscarinic-1 receptors in prelimbic prefrontal cortical neurons of adult male mice

**DOI:** 10.1016/j.ibneur.2021.09.005

**Published:** 2021-10-01

**Authors:** Miguel Garzón, Gang Wang, June Chan, Faye Bourie, Ken Mackie, Virginia M. Pickel

**Affiliations:** a Departamento de Anatomía, Histología y Neurociencia, Facultad de Medicina UAM, Madrid 28029, Spain; b Brain and Mind Research Institute, Weill Cornell Medicine, New York, NY 10065, USA; c Department of Psychological and Brain Sciences, Indiana University, Bloomington, IN, USA

**Keywords:** 2-AG, 2-arachidonoyl-glycerol diacylglycerol, ABC, avidin biotin complex, ACSF, artificial cerebrospinal fluid, BSA, bovine serum albumin, CB1Rs, cannabinoid-1 receptors, DAG, diacylglycerol, ∆9-THC, delta-9-tetrahydrocannabinol, EPSC, excitatory postsynaptic current, ETOH, ethyl alcohol, IPSC, inhibitory postsynaptic current, IP3, inositol 1,4,5-trisphosphate, ITI, intertrial interval, LTD, long term depression, M1Rs, muscarinic-1 receptors, NMDA, N- methyl-D-aspartate, PBS, phosphate buffered saline, PLC, phospholipase C, PL-PFC, prelimbic-prefrontal cortex, PD, postnatal day, RMP, resting membrane potential, SA, spontaneous alternation, TS, Tris-buffered saline, Muscarinic-1 receptor, Prelimbic, Prefrontal cortex, Adolescence, Marijuana, Cannabinoid

## Abstract

Long-term cannabis use during adolescence has deleterious effects in brain that are largely ascribed to the activation of cannabinoid-1 receptors (CB1Rs) by delta-9-tetrahydrocannabinol (∆9-THC), the primary psychoactive compound in marijuana. Systemic administration of ∆9-THC inhibits acetylcholine release in the prelimbic-prefrontal cortex (PL-PFC). In turn, PL-PFC acetylcholine plays a role in executive activities regulated by CB1R-targeting endocannabinoids, which are generated by cholinergic stimulation of muscarinic-1 receptors (M1Rs). However, the long-term effects of chronic administration of increasing doses of ∆9-THC in adolescent males on the distribution and function of M1 and/or CB1 receptors in the PL-PFC remains unresolved. We used C57BL\6J male mice pre-treated with vehicle or escalating daily doses of ∆9-THC to begin filling this gap. Electron microscopic immunolabeling showed M1R-immunogold particles on plasma membranes and in association with cytoplasmic membranes in varying sized dendrites and dendritic spines. These dendritic profiles received synaptic inputs from unlabeled, CB1R- and/or M1R-labeled axon terminals in the PL-PFC of both treatment groups. However, there was a size-dependent decrease in total (plasmalemmal and cytoplasmic) M1R gold particles in small dendrites within the PL-PFC of mice receiving ∆9-THC. Whole cell current-clamp recording in PL-PFC slice preparations further revealed that adolescent pretreatment with ∆9-THC attenuates the hyperpolarization and increases the firing rate produced by local muscarinic stimulation. Repeated administration of ∆9-THC during adolescence also reduced spontaneous alternations in a Y-maze paradigm designed for measures of PFC-dependent memory function in adult mice. Our results provide new information implicating M1Rs in cortical dysfunctions resulting from adolescent abuse of marijuana.

## Highlights

• Cholinergic M1 receptors are located on dendritic plasma and cytoplasmic membranes of in the PL-PFC of adult Mice.
• Repeated administration of ∆9-THC during adolescents impacts the dendritic density of M1Rs in the PL-PFC of adults.
• Dendritic profiles expressing M1 receptors receive input from inhibitory- and excitatory-type terminals containing CB1Rs.
• Chronic ∆9-THC in adolescence attenuates muscarinic evoked hyperpolarization and increases the firing rate of PL-PFC neurons.
• Adolescent ∆9-THC reduces PFC-dependent memory function in adult mice.

## 1 Introduction

Excessive consumption of marijuana’s major psychoactive compound ∆9-THC can disrupt endocannabinoid signaling by chronic occupancy of Gi-coupled CB1Rs, the primary cannabinoid receptor subtype in brain (Rubino et al., 2015, Meyer et al., 2018). Both acute and long-lasting neurocognitive deficits in memory and executive control in humans have been observed following the escalating intake of ∆9-THC through increased recreational use of marijuana in adolescence (Jager et al., 2010, Mooney-Leber and Gould, 2018). This effect also has been shown experimentally by administration of ∆9-THC in female (Rubino et al., 2015) and male rats (Renard et al., 2016). However, there is less information available on the long-term behavioral and neuroanatomical consequences of ∆9-THC administration in adolescent male mice, which differ substantially from females in their delayed maturation and susceptibility to the adverse effects of marijuana (Burgdorf et al., 2020, Long et al., 2013).

Cholinergic inputs and muscarinic-1 receptors (M1Rs) arise early during postnatal development of the frontal cortex (Lee et al., 1990, Mechawar and Descarries, 2001) where their interactions with the endocannabinoid system are major contributors to PFC-dependent learning and memory (Acquas et al., 2001, Gessa et al., 1998, Verrico et al., 2003). G(q)-coupled M1Rs are the most prevalent of the five known muscarinic receptor subtypes in the prelimbic (PL)-PFC (Jiang et al., 2014). Activation of M1Rs can enhance EPSCs and reduce IPSCs in a CB1R-dependent fashion in pyramidal neurons within the cerebral cortex (Nunez et al., 2012, Whalley and Constanti, 2006). This inhibition results from CB1R-mediated retrograde signaling by 2-AG, an endocannabinoid generated from DAG in response to depolarization and/or M1R stimulation (Kurowski et al., 2015).

The sustained dendritic release of 2-AG prolongs the activation of presynaptic CB1 receptors, which include LTD of glutamatergic synapses on layer V principal neurons in the PL-PFC (Sugiura et al., 2002, Verrico et al., 2003). We have shown previously that repeated systemic administration of escalating doses of Δ9-THC through adolescence results in a persistent decrease in the plasmalemmal density of NMDA GluN1 subunits in large dendrites, without contact from CB1R-containing axon terminals in the PL-PFC of adult male mice (Pickel et al., 2020). These mice showed a significant reduction in social interactions with little impairment in attention/memory tasks that are potently affected by systemic administration of scopolamine, a M1R-selective antagonist (Chudasama et al., 2004, Presburger and Robinson, 1999). Together, these observations suggest that enduring cognitive and attentional dysfunctions resulting from chronic cannabis exposure during adolescence are linked to a decrease in expression and function of M1Rs in PL-PFC output neurons of adult male mice. We propose to test this hypothesis by using electron microscopic immunocytochemistry, current clamp recording and behavioral testing in C57BL\6J male mice. Attention and PFC-mediated spatial working memory will be evaluated by Y-maze behavioral tests (Ukai et al., 1995, Zhang et al., 2021) in adult mice that receive either vehicle or Δ9-THC through adolescence. Together, this research will fill the gap in knowledge of the long-lasting effects of cannabis on the developing brain in the postnatal period of adolescence when there is active synaptic pruning and synaptogenesis controlled in part by CB1R-mediated signaling (Hurd et al., 2019, Renard et al., 2016).

## 2 Materials and methods

### 2.1 Animals and drug treatment

All experimental procedures were carried out in accordance with the National Institutes of Health Guidelines for the Care and Use of Laboratory Animals and were approved by the Institutional Animal Care and Use Committees (IACUC) at Weill-Cornell Medical College. C57BL/6J male mice were obtained commercially from Jackson Laboratory (Bar Harbor, ME). The mice were reared in groups of 4–5 mice/cage from weaning at postnatal day (PD) 21 to young adulthood at PD 70 (Spear, 2000). C57BL/6J mice are chosen because they are readily available and well characterized with respect to the behavioral effects of ∆9-THC (Laaris et al., 2010, Wise et al., 2011). All animals were kept in a temperature and humidity-controlled environment and maintained with HEPA-filtered air on a 12-h light/dark cycle. Food and water were available ad libitum.

Nitrogen gas was used to evaporate the ethanol from a solution containing ∆9-THC (100 mg/ml), which was provided by the Drug Supply Program of the National Institute on Drug Abuse (Bethesda, MD, USA). The ∆9-THC residue was dissolved using a modification of that described by Burston et al., (2010) in which the residue is briefly heated at < 100 degrees centigrade in 0.9% NaCl (saline) solutions containing 4%, 8% or 14% Tween 80. These were respectively used to prepare 2.5, 5.0, and 10 mg/kg doses of ∆9-THC, each of which was administered by once daily intraperitoneal injections on five consecutive days totaling 15 days from PD 28–43, which corresponds to the developmental epoch of early/late adolescence in humans (Spear, 2004). This paradigm was chosen from previous studies indicating its similarity to escalating cannabis use in teenagers (Lopez-Rodriguez et al., 2014). Equal numbers of mice were assigned randomly to ∆9-THC (n = 10) and vehicle controls (n = 10) receiving equal quantities of the vehicle (saline and Tween 80). The mice in each treatment group were housed separately in 3–5 mice/cage through the period of injections, after which they were returned to their home cages where they remained without further injections until adulthood at PD 70, when they were euthanized for experimental tissue processing and further analysis. The prefrontal cortical tissue from mice in each treatment group was analyzed by electron microscopic dual immunolabeling and in vitro current-clamp recording by individuals unaware of whether the mice received vehicle or ∆9-THC. The brain tissues from adult mice pre-treated with either vehicle or ∆9-THC during adolescence were co-processed for (1) electron microscopic dual immunolabeling of the CB1R and M1R, or (2) whole-cell current-clamp recording in slice preparations used to determine whether repeated adolescent administration of ∆9-THC alters the M1R induced depolarization of layer III-V pyramidal neurons (Cass et al., 2014).

### 2.2 Tissue preparation for electron microscopic immunolabeling

Ten adult mice receiving repeated injections of vehicle (n = 5) or ∆9-THC (n = 5) during adolescence were deeply anesthetized by intraperitoneal injection of sodium pentobarbital (150 mg/kg) and subjected to vascular perfusion with 4% paraformaldehyde in 0.1 M PB saline, pH 7. The aldehyde-fixed brains were then removed from the cranium and cut in 40 µm thick sections using a Leica Vibratome (Leica Microsystems, Bannockburn, IL, U.S.A.). Coronal sections through the PL-PFC from vehicle and ∆9-THC recipient mice were collected at 1.6 mm anterior to Bregma (Hof et al., 2000). These sections were incubated for 24 h at room temperature in a solution containing both CB1R and M1R antibodies (Alomone Labs, Jerusalem, Israel Cat # AMR-001).

#### 2.2.1 Dual immunoperoxidase and immunogold labeling

The CB1R was identified using an affinity-purified polyclonal antibody raised in guinea pig against a glutathione S-transferase fusion protein containing the C-terminus [residues 401–473) of rat CB1R (Berghuis et al., 2007). This antibody has been shown to have no immunoreactivity in CB1R knockout mice (Fitzgerald et al., 2012, Katona et al., 2006, Pickel et al., 2012). Rabbit polyclonal antibody directed against the third intracellular loop of the human M1 muscarinic receptor**,** Anti-CHRM1 Antibody (#AMR-001) was purchased from Alomone Labs, Jerusalem Israel. The antibody was affinity purified and shown to recognize M1R from human, mouse, and rat samples. Specificity was shown by absence of immunolabeling in tissue from CHRM1 knock out mice. The production and characterization of this antibody is the same as that originally developed by (Levey et al., 1991). The M1R antibody recognized a single band corresponding to the molecular weight predicted for M1R but no other muscarinic receptors (Oda et al., 2018).

The guinea pig CB1 and rabbit M1R antisera were used for immunolabeling at respective dilutions of 1:1000 and 1:200, which were prepared in TS; pH 7.6 containing 0.1% bovine serum albumin (BSA; Sigma-Aldrich, St. Louis, MO). Following incubations with the primary antisera, the PFC tissue sections were washed in TS and processed for dual immunoperoxidase and immunogold labeling using a modification (Milner et al., 2011) of the method developed by Chan et al., (1990). For immunoperoxidase labeling of the CB1R, the tissue was first incubated for 30 min in a TS and 0.1% (BSA) containing 1:200 dilution of donkey anti-guinea pig biotinylated IgG (Jackson Immunoresearch, West Grove, PA). This was followed by washing in TS and a one-hour incubation in avidin biotin complex (Vector ABC Elite kit; (Vector Labs, Burlington, CA). A six-minute reaction in 3, 3′-diaminobenzidine (DAB, Sigma-Aldrich, St. Louis, MO) with 0.1% hydrogen peroxide was used to visualize the peroxidase reaction product. Following the DAB reaction, the tissue sections were incubated for 2.5 h in a 1:25 dilution of Ultrasmall gold goat anti-rabbit IgG (Electron Microscopy Sciences). The tissue was then post-fixed in 2% glutaraldehyde in 0.01 M PBS solution for 10 min and washed in 0.1 M PB prior to being transferred to 0.2 M citrate buffer in preparation for silver-intensification by an 8-minute incubation in a solution provided in the Structure Probe Inc Silver enhancement kit (SPI Supplies, West Chester, PA). The silver-intensified sections were rinsed sequentially in 0.2 M citrate buffer and 0.1 M PB, and then post-fixed in 2% osmium tetroxide in 0.1 M PB before embedding in Epoxy resin in preparation for electron microscopic analysis using conventional methods (Milner et al., 2011).

### 2.3 Image analysis

Electron microscopic images were analyzed from ultrathin sections taken from middle layers of the PL-PFC extending from 1000 to 2000 µm from the pial surface in adult mice that received once daily injections of vehicle or ∆9-THC through adolescence (PD 28–43). Two plastic-embedded slices of tissue were sampled from 10 adult mice receiving either vehicle (n = 5) or ∆9-THC (n = 5) during adolescence. The density of unlabeled and immunolabeled neuronal profiles was calculated from 50 images captured at a magnification of 18,500X equally from each of the ten mice. Immunoperoxidase labeling for CB1R was regarded as positive when the electron dense precipitate in individual profiles was greater than that seen in other morphologically similar profiles in the neuropil. Structures containing one or more immunogold-silver deposits were identified as immunolabeled for M1R. This approach avoids experimental bias against smaller structures that generally have fewer M1R gold particles than larger dendrites but may also contribute to false positive results. Since spurious gold-silver deposits were rarely seen overlying structures not known to contain M1Rs, this is unlikely to be a complication.

Because size and shape are intimately linked to function (Kopec and Malinow, 2006), we used MCID Analysis software, Version 7.0 (Focus Ltd, Cambridge, UK) to measure the mean diameter, major axis and minor axis length, perimeter, area, and form factor of immunolabeled neuronal profiles. A cluster analysis of the mean diameter of unlabeled and M1R-labeled dendritic profiles (shafts and spines) was performed to statistically separate dendritic profiles by size, since large and small branches of cortical neurons vary in their CB1R-receptor distributions (Pickel et al., 2020). JMP Statistical Discovery from SAS (Cary, NC) was then used to determine statistically significant (*p* < 0.05) differences in M1R gold particle density in dendritic profiles of each size range in the PL-PFC of vehicle or ∆9-THC pre-treated mice. Two-way ANOVA (drug x size) JMP statistics was also used to compare M1R-labeled dendritic profiles in each of three (small, medium, and large) sizes. These were further separated by presence or absence of synaptic input form terminals containing CB1 and/or M1 receptors.

Microscopic illustrations were prepared by importing digital images into Adobe Photoshop (CS4) and Powerpoint (Microsoft Office, 2016) to enhance contrast, prepare composite plates, and add lettering. Labeled neuronal and glial profiles were defined using the nomenclature of Peters et al. (1991).

### 2.4 Whole-cell current-clamp recordings

Whole-cell configuration recordings were made in pyramidal neurons identified by their firing properties in layers III-V of the medial PL-PFC in brain slices from adult male mice that received intraperitoneal (ip) injections of vehicle (n = 5) or ∆9-THC (n = 5)through adolescence (see above section on animals). For this, the mice were anesthetized with 2% isoflurane, and their brains rapidly removed and immersed into ice-cold sucrose (s)-ACSF (see Park et al., 2014). After the whole-cell configuration was formed, Mg^2+^-free lactic acid-ACSF was used to super fuse the slices. The access and membrane resistances were tested and continuously monitored through the recording. Only those cells in which access resistance was stable (change <10%) were included in the data analysis. Stable baseline recordings of the resting membrane potential (RMP) were achieved before local application of the buffer control. The EPSC was recorded on pyramidal neurons of the PL-PFC by 0.1 Hz stimuli through an aCSF-filled glass electrode dorsal to the recording site. Bath application of a non-selective M1R agonist, carbamyl-β-methylcholine chloride (CBM), was applied over a dose range of 100–300 µM.

*Data Analysis:* Electrophysiological data is expressed as means ± SEM in recorded cells from at least 5 slices per treatment group. A two-way ANOVA was used to compare the difference of the drug-induced effect on the RMP, frequency of spikes, and amplitude of EPSCs with a *p* < 0.05 considered statistically significant. RMP and frequency of spontaneous spikes were analyzed offline using Window pClamp 10.3 (Molecular Devices) and MiniAnalysis (Synaptosoft), respectively. The liquid junction potential was corrected during offline analysis.

### 2.5 Behavioral testing

Behavioral testing was conducted in a subgroup of young adult (PD70) male mice that received either vehicle (n = 5) or Δ9-THC (n = 8) during adolescence (PD 28-43) and were behaviorally tested 1–2 days prior to sacrifice on PD70. To avoid potential confounding effects of behavioral measures, these tests were done in a separate group of mice from those used for electron microscopic immunolabeling or current clamp recording. Animals were habituated to the testing room approximately one hour prior to beginning behavioral testing, which occurred during the light phase of the light/dark cycle. The apparatus used for testing was cleaned thoroughly with 70% EtOH and dH_2_0 between mice to remove olfactory cue biases. Unpaired *t*-tests (GraphPad Prism, version 8) were conducted for all between-group comparisons for each of the parameters measured with a *p* < 0.05 considered statistically significant.

*2-Trial Y-maze Task* (Cruz Hernandez et al., 2019) was used to assess short-term spatial recognition memory and novelty exploration using a modified protocol developed by Park et al. (2008). During trial one (training phase), mice were placed into one of two “start arms” (either A or B) while one arm of the Y-maze designated as the “novel arm” (arm C) was closed off with a guillotine door. Subjects could freely explore the remaining two arms of the maze for 5 min. Upon completion of training, mice were immediately removed from the maze and returned to their home cage while the guillotine door blocking arm C was removed. After an approximately 150 min intertrial interval, mice were placed in their designated start arm and were given 5 min to explore all three arms of the maze, during which time, the number of entries into the novel arm and the percentage of SA were recorded. An alternation occurred when a mouse entered a different arm of the maze in each of 3 consecutive arm entries. The percentage of SA was then calculated by dividing the total number of alternations by the total number of arm entries, multiplied by 100. Mice with intact short-term recognition memory remember which arms have been previously explored, and over the course of the test they tend to visit a novel arm more frequently than the other arms. A reduction in SA is an indication of impaired spatial working memory, which may reflect an inability to recall spatial cues formed about the maze environment at the time of memory acquisition (i.e. training). Distance traveled, mean speed, and total number of arm entries were also measured as a quality control check to assess general activity levels. Mice that completed less than 9 total arm entries in the SA test were considered outliers and removed from the dataset. Behavior was video recorded using a camera positioned directly above the maze and quantitative parameters used for analysis were recorded using AnyMaze software (Stoelting, Wood Dale, IL).

## 3 Results

Our results show M1R-immunogold particles located mainly in dendritic profiles and abundant CB1R immunoperoxidase labeling in axon terminals in the PL-PFC of adult male mice in both vehicle and Δ9-THC treatment groups. However, repeated adolescent administration of Δ9-THC resulted in size-dependent changes in the density of M1Rs in dendrites and dendritic spines in the PL-PFC of adult mice. Current clamp recordings also revealed that adolescent pre-treatment with Δ9-THC attenuates M1R-mediated hyperpolarization in PL-PFC neurons. This finding is consistent with our behavioral results showing that chronic adolescent exposure to Δ9-THC decreases spontaneous alternation, which may reflect a decrease in M1R-mediated excitation or increase in CB1R-mediated inhibition in principal neurons in the PL-PFC.

### 3.1 Dendritic M1R distribution in vehicle and Δ9-THC pre-treated mice

**Fig. 1:**
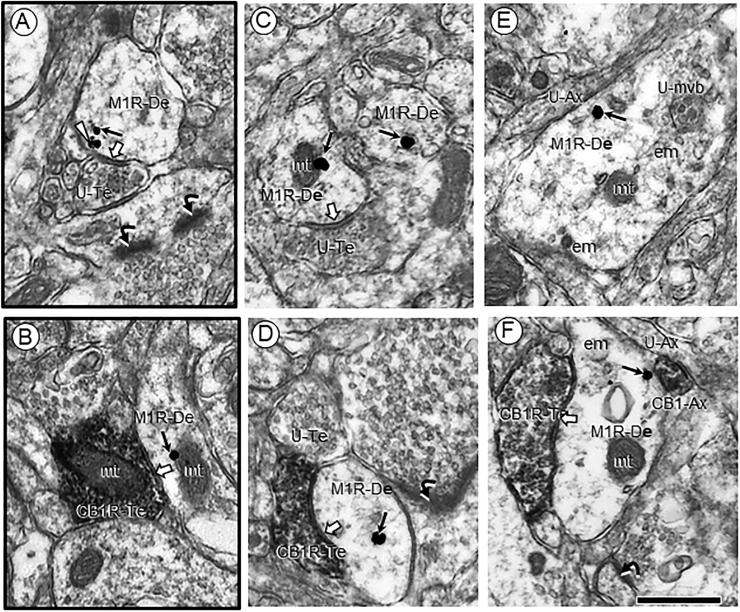
M1R immunogold in the cytoplasm of small dendritic shafts receiving synaptic input from unlabeled (A–E) and CB1R immunoperoxidase labeled (B–F) axon terminals within the PL-PFC of adult male mice. These mice received 14 consecutive daily injections of vehicle (A,B black border) or ∆9-THC (C,E no border) through adolescence. Panel A shows, M1R immunogold (small arrows) in the cytoplasm and in contact with the perisynaptic plasma membrane contacted by an unlabeled terminal forming a symmetric synapse (block arrows) like the junction formed by a CB1-labeled terminal in B. Panels C-D and E-F respectively show unlabeled and CB1R-labeled terminals presynaptic to dendritic profiles with a mainly cytoplasmic distribution of M1R gold particles in the PL-PFC of mice that received ∆9-THC during adolescence. M1R-De = Immunogold-labeled dendrite; CB1R-Te = immunoperoxidase CB1R-labeled axon terminal; white block arrows = symmetric synapses; U-Ax = unlabeled axon; CB1-Ax = CB1R-labeled axon; curved black arrows = asymmetric excitatory-type synapses; small black arrows = cytoplasmic M1R gold particles; white arrowheads = perisynaptic plasmalemmal M1R immunogold; U-Te = unlabeled terminal; U-mvb = unlabeled multivesicular body; mt = mitochondrion = Scale bar = 500 nm.

**Fig. 2:**
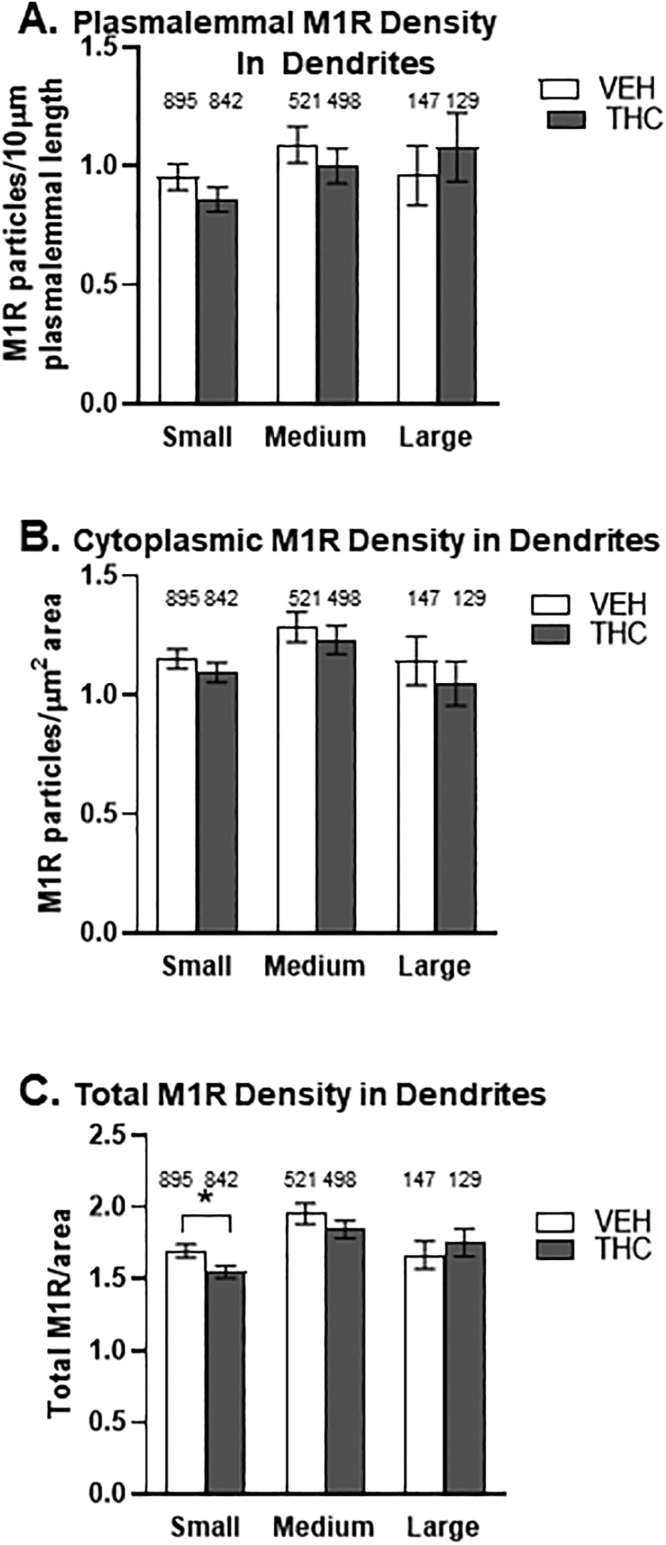
Bar graphs showing a significant reduction in M1R immunogold in PL-PFC dendrites of adult mice receiving Δ9-THC (THC) as adolescents. As compared to vehicle (VEH) mice, animals receiving Δ9-THC had a lower mean density of M1R immunogold particles on the plasma membrane (A) and in the cytoplasm (B) of small, but not medium or large sized dendrites of adult mice chronically exposed to Δ9-THC during adolescence. In (C) the reduction in M1R density in small dendrites of Δ9-THC pretreated mice is statistically significant only when including the total of both plasmalemmal and cytoplasmic labeling (*p* < 0.05; Welch’s correction for unequal variance). Values on top of the bars depict absolute number of dendrites in each category.

In both vehicle and Δ9-THC pretreated mice, M1R immunogold was localized to non-synaptic and peri-synaptic plasma membranes and to cytoplasmic organelles in dendritic shafts (Fig. 1). When clustered by size, these shafts displayed mean diameters in the range of 0.46±0.09 µm. These include small (n = 1737) at 0.46±0.09 µm, medium (n = 1019) at 0.76±0.11 µm, and large (n = 276) at 1.30±0.26 µm. THC-injected mice displayed a significant decrease (M=1.55) in the total density of M1- immunogold particles in small dendrites in the PL-PFC compared to VEH-treated mice (M = 1.7). (t(1734) = 2.4, *p* = 0.0166; Welch’s correction for unequal variance). No significant between group differences (*p* > 0.05) were seen in medium or large dendritic shafts in the PL-PFC of adult mice pre-treated with Δ9-THC compared to vehicle (Fig. 2).

The M1R immunogold particles were localized to tubulovesicles, multivesicular bodies and mitochondria within the cytoplasm of dendritic shafts, where many other similar types of vesicles were without immunogold labeling. The greater prevalence of unlabeled compared to M1R-labeled multivesicular bodies, which are important mediators of intracellular trafficking of multiple G-protein coupled receptors (Bloch et al., 2003) was quantitatively confirmed. From a total of 383 multivesicular bodies identified in PL-PFC dendrites, only 0.26% (n = 1) in the vehicle and 1.83% (n = 7) in the Δ9-THC pretreated group showed M1R immunogold labeling. In contrast, unlabeled multivesicular bodies comprised 42.04% (n = 161) in the vehicle and 55.87% (n = 214) in PL-PFC dendrites of mice that received Δ9-THC during adolescence. The increase in M1R-labeled and unlabeled multivesicular bodies in PL-PFC dendrites of Δ9-THC pretreated mice suggests heightened intracellular mobilization of many GPCRs other than M1Rs. M1R-labeled MVBs are proportionally greater in number than unlabeled MVBs in the PL-PFC dendrites of Δ9-THC pretreated mice, but these differences were not statistically significant (Fisher's exact test, two sided; *p* > 0.05).

### 3.2 Size- and treatment-specific M1R distribution in dendritic spines

**Fig. 3:**
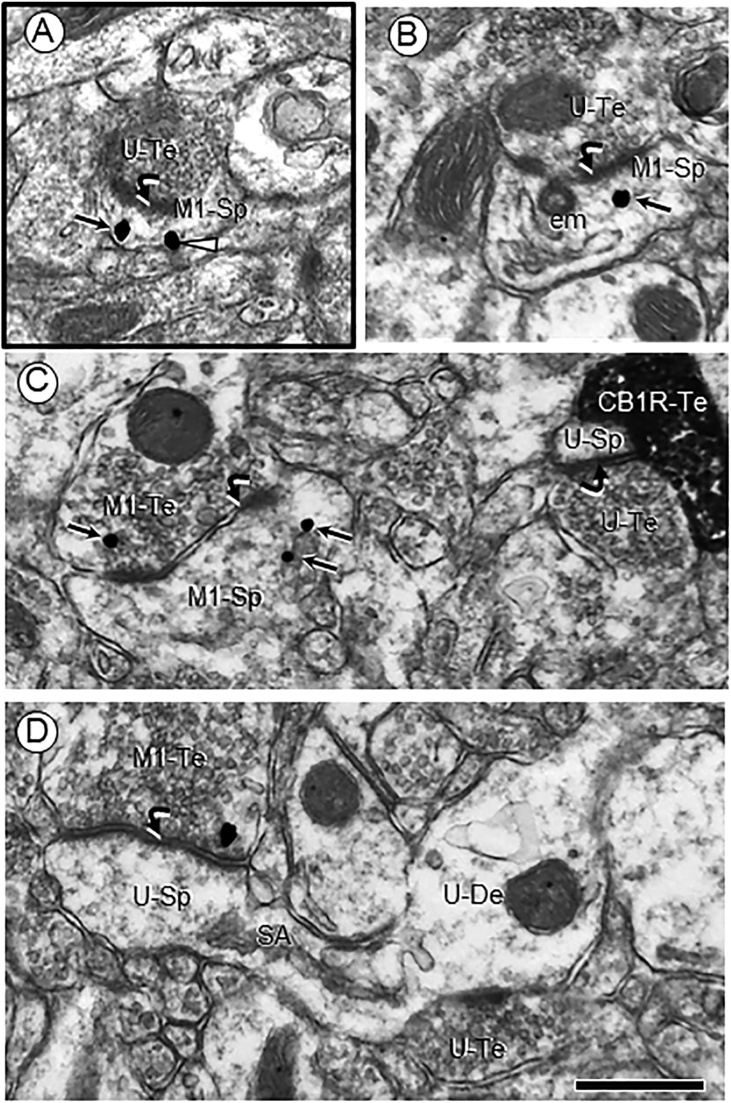
Separable locations of M1R immunogold and CB1R-immunoperoxidase labeling at axo-spinous synapses in the PL-PFC of adult male mice receiving vehicle (A) or Δ9-THC (B–D) as adolescents. In A, M1R immunogold is located on (arrowhead) and near the extrasynaptic plasmalemma of a small dendritic spine (M1-Sp) that receives excitatory-type input from an unlabeled axon terminal (U-Te). Panel B shows M1R immunogold in the cytoplasm near endomembranes (em) beneath an asymmetric synapse formed by an unlabeled axon terminal. M1R gold is seen in axon terminals forming asymmetric synapses with large dendritic spine heads containing M1R immunogold particles (M1-Sp in C) or without immunoreactivity (U-Sp in D). In the upper right corner of panel C, a CB1R peroxidase labeled terminal apposes an axospinous synapse between unlabeled profiles. Small arrows = cytoplasmic M1R immunogold; curved arrows = asymmetric synapses; U-Te = unlabeled axon terminal (U-Te). Scale bar = 500 nm.

The M1R immunogold particles seen in small dendritic spines within the PL-PFC of adult mice receiving either vehicle or Δ9-THC as adolescents were prevalent on plasma membranes distant from asymmetric, excitatory-type synapses formed by unlabeled or M1R-labeled axon terminals (Fig. 3A). In larger dendritic spines, M1R immunogold was primarily associated with endomembranes, some of which were in continuity with the postsynaptic membrane specialization (Fig. 3B–D).

Quantitative comparison of the plasmalemmal and cytoplasmic analysis of a total of 296 M1R- labeled dendritic spines from the PL-PFC showed no significant difference between vehicle and Δ9-THC-pretreated mice (*p* > 0.05; Welch’s correction for unequal variance). When the k means cluster of spine area and diameter was used to separate M1R-labeled spines by size, the smaller spines were almost twice as prevalent as the larger dendritic spines in both vehicle (n = 133, small; and n = 65, large) and Δ9-THC (n = 145, small; n = 80, large) treatment groups. However, only the larger dendritic spines in the Δ9-THC pretreatment group had a significantly (*p* = 0.0367; Power = 0.5547) higher M1R cytoplasmic density compared to the vehicle controls. There was also a notable, but non-statistically significant decrease in the density of M1R immunogold on the plasma membrane of large dendritic spines. This is consistent with increased internalization and/or decreased plasmalemmal insertion of M1Rs in large spines within the PL-PFC of adult mice receiving repeated injections of Δ9-THC during adolescence.

### 3.3 M1R gold distribution in non-synaptic and synaptic axonal profiles

**Fig. 4:**
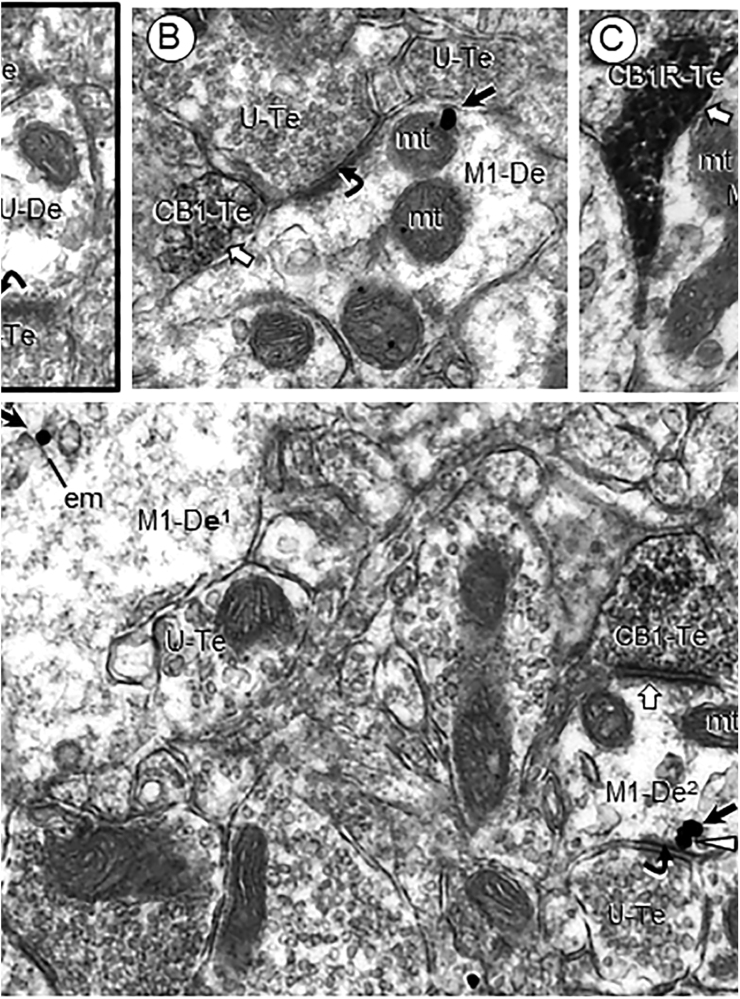
Subcellular localization of M1R immunogold and CB1R-immunoperoxidase labeling at axo-dendritic synapses in the PL-PFC of adult mice receiving vehicle (A) or Δ9-THC (B–D) as adolescents. Panel A shows a dually labeled axon terminal (Du-Te) with one M1R gold particle on the presynaptic membrane of an axon terminal containing peroxidase immunoreactivity for CB1R and one large mitochondrion (mt). The symmetry (block arrow) of this inhibitory-type junction contrasts with the thickened asymmetric synapse (curved arrow) formed by an unlabeled terminal (U-Te) convergent on the same unlabeled dendritic profile (UDe). Panel B shows a similar convergent labeling of a single CB1R-immunoreactive terminal and an excitatory-type synapse on a dendrite (M1R-De) in which the M1R gold is aligned on outer membranes contacted by an unlabeled terminal. Cytoplasmic M1R immunogold is associated with a mitochondrion (mt) and on a multivesicular body (mvb) in B and C, respectively. In D, M1R immunogold is localized to endomembranes (em) near the plasma membrane in a dendritic profile (M1-De^1^) and to the perisynaptic plasma membrane in a second dendrite (M1-De^2^). One gold particle (arrowhead) opposes the membrane specialization of an asymmetric synapse formed by an unlabeled axon terminal. This dendrite also receives convergent input from an axon terminal containing CB1R peroxidase reaction product. Cytoplasmic M1R gold is shown by small black arrows in all images. Curved arrows = asymmetric synapses; white block arrow = symmetric synapses. Scale bar = 500 nm.

M1R immunogold particles were identified in numerous vesicle-filled axonal profiles in the PL-PFC of adult mice. Of the total observed M1R-labeled axonal profiles, the majority, 72% of 366 in vehicle and 64% (n = 471) in THC treatment groups were without recognizable synaptic membrane specializations. The remainder formed symmetric inhibitory-type synapses or asymmetric excitatory-type synapses with dendrites and dendritic spines some of which expressed M1R labeling (Table 1). M1R immunogold was rarely seen on and near the presynaptic membrane specialization in axon terminals forming asymmetric synapses with dendritic spines (Fig. 3 and Table 1). These dendritic spines were largely without detectable M1R immunogold and were substantially more prevalent in the PL-PFC of Δ9-THC pretreated mice (Table 1). In contrast with dendritic spines, M1R immunogold was rarely seen in axon terminals forming asymmetric synapses on dendritic shafts even though these dendritic profiles received convergent input from many unlabeled terminals forming this type of synaptic specialization (Fig. 4). The M1R- containing terminals also infrequently formed symmetric inhibitory-type synapses. These included those that colocalized CB1Rs and terminated on unlabeled dendrites.

**Table 1:** M1R-labeled axon terminals forming either symmetric or asymmetric synapses with dendritic profiles defined by their expression of M1R in the PL-PFC of adult male mice pre-exposed to vehicle or Δ9-THC during adolescence.

M1R-Terminals	Vehicle N = 101		△9-THC N = 193	
**Symmetric Synapse**	*% M1R*	*% Non-M1R*	*% M1R*	*% Non-M1R*
*Dendritic Shafts*	–	2.9% (3)	2.6 (5)	10.4 (20)
*Dendritic Spines*	–	–		2.6 (5)
**Total**	2.9%		2.6%	13.0%
				
**Asymmetric Synapse**	*%M1R*	*% Non-M1R*	*% M1R*	*%Non-M1R*
*Dendritic Shafts*	2.0 (2)	19.8 (20)	10.4 (20)	5.2 (10)
*Dendritic Spines*	6.9 (7)	67.3 (68)	4.7 (9)	2.4 (24)
**Total**	8.9%	87.1%	15.0%	69.4%

Total area sampled 43,350 µm^2; 21,675 µm2 each for vehicle and Δ9-THC N = total number of M1R-labeled axonal profiles in synaptic contact with dendritic profiles containing or not MIR-immunoreactivity. Numbers in parenthesis indicate percentages of total N in each category.^

### 3.4 CB1 receptors presynaptic to dendritic profiles containing M1R immunogold

Immunoperoxidase labeling for CB1Rs was seen in axon terminals forming mainly, but not exclusively symmetric contacts with M1R-labeled and unlabeled dendritic shafts of varying sizes in the PL-PFC of both vehicle and Δ9-THC pretreated mice (Fig. 1B–E; and Table 2). The CB1R-labeled terminals forming excitatory-type synapses often contacted the smaller M1R containing dendrites and dendritic spines regardless of whether the mice received vehicle or Δ9-THC during adolescence, although they were more prevalent in the latter group (Table 2).

**Table 2:** CB1R-terminals forming symmetric or asymmetric synapses with M1R-labeled or unlabeled dendritic profiles in the PL-PFC of adult mice that received vehicle or Δ9-THC during adolescence.

CB1R terminals	Vehicle N = 291		∆9-THC N = 729	
**Symmetric Synapse**^a^	*% M1R*	*% Non-M1R*	*% M1R*	*% Non-M1R*
*Dendritic Shaft*	44.0 (139)	41.6 (121)	18.5 (135)	44.6 (325)
*Dendritic Spine*	2.0 (6)	2.0 (6)	10% (73)	1.7 (85)
**Total**	49.8 (145)	43.6	28.5%	56.3%
				
**Asymmetric Synapse**	*% M1R*	*% Non-M1R*	*%M1R*	*%Non-M1R*
*Dendritic Shaft*	0.7 (2)	3.4 (10)	0.5 (4)	0.8 (6)
*Dendritic Spines*	1.7 (5)	0.7 (2)	6.9 (50)	7.0 (51)
**Total**	2.4%	4.1%	7.4%	7.8%

Total area sampled 43,350 µm^2^; 21,675 µm^2^ each for VEH and Δ9-THC. Numbers in parenthesis indicate percentage of total N in each category.

a Includes contacts with and without symmetric membrane specializations.

### 3.5 Consequences of Δ9-THC pretreatment on response of PL-PFC neurons to muscarinic stimulation

**Fig. 5:**
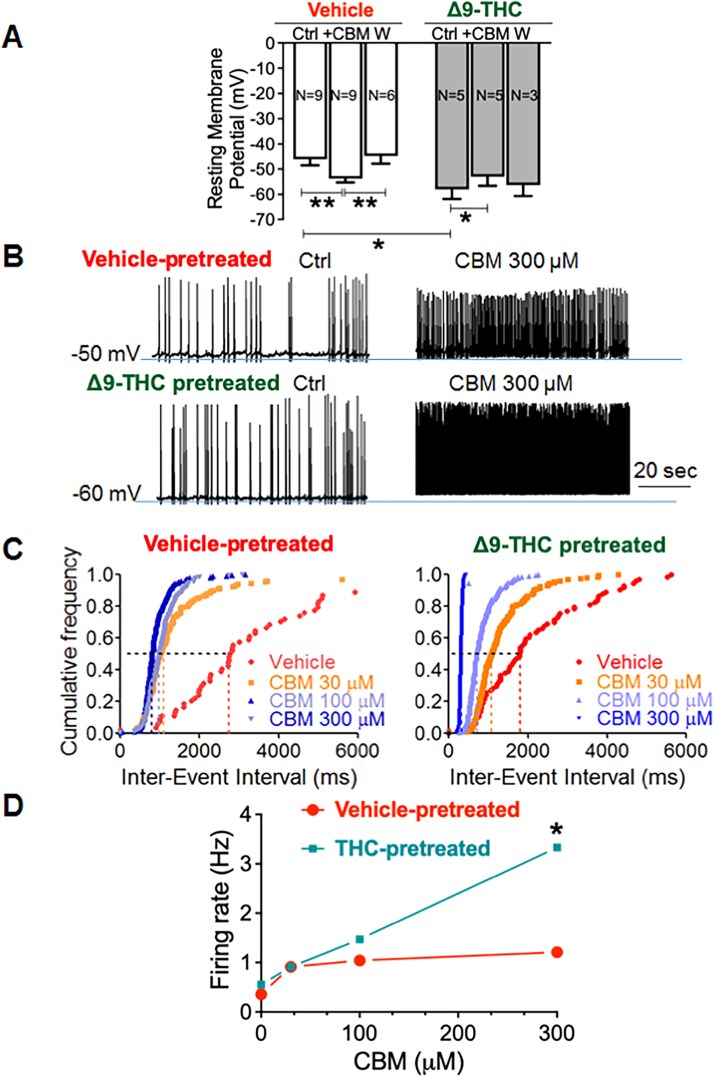
Whole-Cell Current-Clamp Recording of PL-PFC neurons after muscarinic agonist application on brain slices from adult male mice receiving either vehicle or Δ9-THC as adolescents. Recordings in A demonstrate that bath application of 300 µM of a selective muscarinic agonist, carbamyl-beta-methylcholine (CBM) has a significant hyperpolarizing effect) that is attenuated by adolescent pretreatment with Δ9-THC (*p* = 0.0376, F (1.713,8.223) = 5.241. Panel B shows recording from PL-PFC neurons of vehicle and Δ9-THC-pretreated mice. The panel also shows an effect of CBM on the membrane potential of PL-PFC neurons from mice in both vehicle and Δ9-THC-pretreated mice. In this panel W indicates application of wash. In C-D CBM is shown to dose-dependently increase the firing rate in PL-PFC neurons from vehicle and Δ-THC-pretreated mice. Clampfit 10 programs were used to analyze the firing data. ANOVA, *p < 0.05; ** *p* < 0.01.

Whole-cell current-clamp recording in slice preparations through the PL-PFC showed that bath application of 300 µM carbamyl-beta-methylcholine (CBM) had a significant hyperpolarizing effect that was attenuated by adolescent pretreatment with Δ9-THC (Fig. 5A) (*p* = 0.0376, F (1.713,8.223) = 5.241). The diminished hyperpolarizing effect by CBM lead to changes in the membrane potential that favored an increase in the firing rate in PL-PFC neurons of mice receiving Δ9-THC. In these mice, the application of CBM, dose-dependently increased the firing rate in PL-PFC neurons from Δ9-THC-pretreated mice (Fig. 5C and D) in comparison to vehicle-pretreated mice. Further studies are needed to determine if these effects are mediated by M2/M4, M1 receptors, endocannabinoids, or another mechanism.

### 3.6 Long-term effect of chronic adolescent Δ9-THC exposure on working memory

**Fig. 6:**
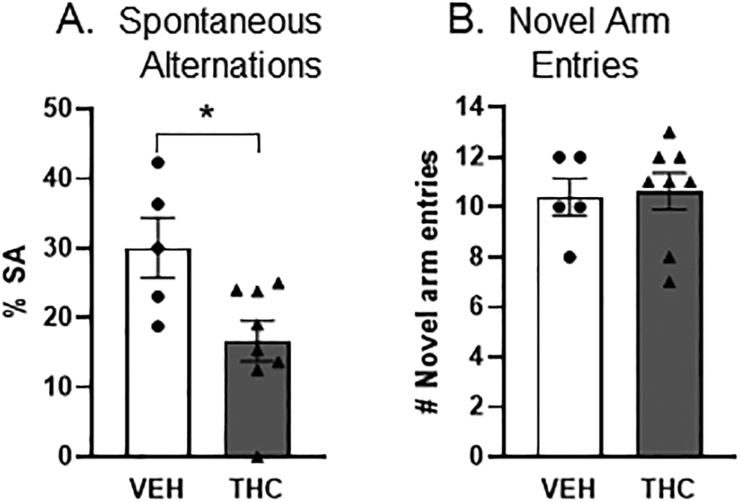
Decreased ratio of spontaneous alternations (SA) but not of novel arm entries in adult mice receiving Δ9-THC as adolescents. In A bar graph shows a significant effect (t(11) = 2.68, *p* = 0.02) of chronic adolescent Δ9-THC exposure on spatial woraxonal profiles in synaptic contact king memory. Adult mice pre-treated with Δ9-THC in adolescence displayed a significant reduction (M = 16.67) in the mean percentage of SA completed within the Y-maze compared to vehicle (VEH)-injected mice (M = 30.1). There were no significant differences between vehicle and Δ9-THC pre-treated mice in the mean number of novel arm entries made within the Y-maze (Panel B; *p* > 0.05).

Δ9-THC pre-treated mice displayed a significant reduction (Fig. 6A; t (11) = 2.68, *p* = 0.02) in the mean percentage (M= 16.67) of spontaneous alternations completed within the Y-maze compared to mice pre-treated with vehicle (M = 30.1). There were no significant treatment-specific differences (*p* > 0.05) between adult mice receiving either vehicle or Δ9-THC during adolescence in the mean number of novel arm entries made within the Y-maze (Fig. 6B). Locomotor activity as measured by the mean distance traveled, mean speed, or total number of arm entries did not differ significantly (*p* > 0.05) between treatment groups, indicating intact motor function. These results suggest that chronic exposure to Δ9-THC during the vulnerable period of adolescence leads to an impairment of spatial working memory and cognitive function without significantly impacting locomotion or novelty exploration in the context of a Y-maze environment.

## 4 Discussion

Our results provide the first ultrastructural evidence for pre- and post-synaptic distributions of M1Rs in middle layers of the PL-PFC of adult male mice chronically exposed to escalating doses of Δ9-THC through early adolescence. There were no apparent qualitative differences between vehicle and Δ9-THC pretreated mice in the morphological features of neurons expressing M1 or CB1 receptors in the PL-PFC. However, adolescent administration of Δ9-THC produced size-dependent changes in the density of M1R immunogold particles in dendritic shafts and spines. These changes may contribute to the diminished M1R-mediated hyperpolarization in PL-PFC neurons and PFC-dependent memory impairment resulting from chronic adolescent administration of ∆9-THC. These results are discussed along with their implications for understanding the role of the muscarinic and cannabinoid receptor systems in synaptic plasticity contributing to cognitive and attentional defects produced by excessive marijuana use during adolescence (Dumitriu et al., 2010, O'Shea et al., 2006, Renard et al., 2016).

### 4.1 M1R distribution in dendritic shafts and spines impacted by repeated adolescent administration of Δ9-THC

We have shown that M1Rs are principally localized to non-synaptic plasma membranes in middle-layer PL-PFC dendrites. These results confirm and extend earlier studies showing that the dendritic distribution of these receptors favors activation by acetylcholine release from varicosities at variable distances from the synapse (Yamasaki et al., 2010). Consistent with earlier results, the majority are likely pyramidal cells, but may also include inhibitory interneurons although this varies depending upon the cortical layer and subpopulation of neurons examined (Oda et al., 2018).

The present demonstration of a significantly higher cytoplasmic density of M1R immunogold in large, but not smaller, thin dendritic spines in the PL-PFC of Δ9-THC pretreated mice is consistent with earlier evidence that small, thin, and larger mushroom type spines are linked to learning and memory (Bourne and Harris, 2007). The increase in M1R immunogold density in the cytoplasm of large dendritic spines in the PL-PFC of Δ9-THC pre-treated mice suggests that these receptors are resources which function to mobilize to potentiated synapses. The ability of dendritic spines to concentrate Ca^2+^ is important for long-term potentiation that leads to learning and memory storage (Holmes and Levy, 1990).

We also observed M1R immunogold particles on membranous cytoplasmic organelles inclusive of multivesicular bodies in both vehicle and Δ9-THC injected mice. This finding suggests that chronic stimulation of CB1Rs through repeated exposure to Δ9-THC may promote M1R plasma membrane depletion and increased internalization of M1R and other GPCRs via endosomal pathways responsible for receptor trafficking, recycling, and degradation. Ubiquitin-regulated sorting into inner vesicles of these bodies may partially account for the down regulation of many activated signaling proteins (Katzmann et al., 2002). M1Rs are positively coupled to PLC that increases the intracellular levels of IP3 and, importantly, of DAG, which is itself a direct precursor of 2-AG (Rubino et al., 2000). Further studies are necessary to determine whether repeated exogenous administration of cannabinoid (aka THC) that mimics endogenous cannabinoids (i.e., 2-AG) disrupts downstream signaling in this pathway via an abnormal increase in IP3-mediated Ca^+2^ release resulting in excessive neuronal activity.

The present localization of M1R on outer mitochondrial and endomembranous structures in PL-PFC neurons is also consistent with downstream signaling of cAMP and/or PKC to enhance cytoplasmic uptake and release of Ca^2+^ from IP3 sensitive stores (Berridge, 2002, Taylor et al., 2014).

### 4.2 Postsynaptic depolarization-induced enhancement of inhibition

CB1Rs in our study were often strategically placed in axon terminals presynaptic to M1Rs-containing dendritic profiles in the PL-PFC. This suggests that CB1Rs are largely responsible for muscarinic enhancement of retrograde endocannabinoid signaling as has been described previously in the hippocampus, (Ohno-Shosaku et al., 2003). The presynaptic location of CB1Rs opposite dendritic shafts either expressing or lacking M1Rs may differ in the extent to which increased 2-AG is produced in response to Ca^2+^-entry through activated glutamate NMDA receptors (Stella and Piomelli, 2001). Long-term changes in the strength of glutamatergic synapses requires participation of NMDA receptors that are less effective in mediating depolarization in PL-PFC neurons from adult mice earlier exposed to Δ9-THC (Pickel et al., 2020).

Of the CB1R-labeled terminals forming synapses on M1R-containing dendrites, there were proportionally smaller numbers of inhibitory and larger numbers of excitatory-type synapses observed in Δ9-THC pretreated mice compared with vehicle. This difference is consistent with attenuation of the hyperpolarized membrane potential and increase in the firing rate in the Δ9-THC treatment group, an effect that was confirmed by current-clamp recordings in the present study. The reduction in muscarinic hyperpolarization seen in PL-PFC pyramidal cells of Δ9-THC pretreated mice may reflect the induction of an enduring postsynaptic depolarization-induced enhancement of inhibition and/or suppression of excitation (DSI/DSE; Diana and Marty, 2004). This form of synaptic plasticity may underlie the attentional and short-term memory deficits produced by disruption of M1R function in PL-PFC neurons as has been described in hippocampal pyramidal neurons (Dominguez et al., 2017).

The complex mechanism underlying the hyperpolarization seen in patch-clamp recording of cortical neurons in adult mice pretreated with Δ9-THV is not fully understood, but others have shown that CB1 receptor activation by THC induces hyperpolarization by activation of G protein-coupled inward rectifier K^+^ (GIRK) channels (McAllister et al., 1999). Though multiple ion channels are involved in neuronal effects induced by activation of M1 receptor (M1-R), M1-R is responsible for an transient hyperpolarization via the small-conductance Ca^2+^-activated K^+^ (SK) channels (Dasari et al., 2017). The M1-R agonists, including carbamyl-β-methylcholine chloride (CBM), can also enhance the NMDA receptor activity in the central neurons, leading to depolarization and firing. See Pickel et al. (2020) for further discussion of NMDA receptor plasticity resulting from chronic adolescent THC administration. Our results showed that CBM induced the transient hyperpolarization in the vehicle-treated neurons. However, there was no transient hyperpolarization observed in the THC-treated neurons. It is likely that the underlying mechanisms for increased firing in the baseline control condition and depolarization in the presence of CBM may result from synergistic actions of cannabinoids and CBM on the NMDA receptor mediated Ca^2+^ signals (Marino et al., 1998, Netzeband et al., 1999).

### 4.3 Significance

The observation of CB1R in axon terminals forming mainly symmetric inhibitory-type synapses with dendrites confirms and extends previous reports of CB1R location in PL-PFC of adult male rats (Fitzgerald et al. 2013) and mice (Pickel et al., 2020). Our present study provides new insight to the intricate association between M1 and CB1 receptors in the PL-PFC within the context of repeated adolescent administration of Δ9-THC in male mice. Our findings are consistent with earlier evidence that ongoing synaptic pruning during adolescent cortical development is greatly altered by escalating daily cannabis use in humans during the vulnerable period of adolescence (Dow-Edwards and Silva, 2017). However, it is not known whether the described neuroplastic changes of Δ9-THC consumption on muscarinic signaling are distinctive or exclusive for the sensitive period of young adolescence, since thus far no studies of these receptors have been performed to evaluate their distribution in cortical neurons following chronic Δ9-THC administration in later adolescence or in adulthood. Moreover CB1R-dependent changes in synaptic pruning and/or synaptogenesis could underlie the observed increase of synapses after Δ9-THC adolescent exposure (Papariello et al., 2014). To discern between these possibilities, a graded age-related study evaluating variations in synaptic densities, which is out of the scope of the present study, would be needed. Altogether, however our finding supports the conclusion that cognitive dysfunction associated with daily cannabis consumption reflects an imbalance of excitation and inhibition in frontal cortical neurons, an effect which persists into adulthood long after cessation of use (Schweinsburg et al., 2008).

## CRediT authorship contribution statement

**Miguel Garzón:** Conceptualization, Funding acquisition, Investigation, Writing – original draft, Writing – review & editing. **Gang Wang:** Investigation, Methodology, Writing – review & editing. **June Chan:** Investigation, Methodology, Writing – review & editing. **Faye Bourie:** Investigation, Methodology, Writing – review & editing. **Ken Mackie:** Investigation, Methodology, Writing – review & editing. **Virginia M. Pickel:** Conceptualization, Funding acquisition, Investigation, Resources, Supervision, Writing – original draft, Writing – review & editing.
